# Accuracy and practical aspects of semi- and fully automatic segmentation methods for resected brain areas

**DOI:** 10.1007/s00234-020-02481-1

**Published:** 2020-07-20

**Authors:** Karin Gau, Charlotte S. M. Schmidt, Horst Urbach, Josef Zentner, Andreas Schulze-Bonhage, Christoph P. Kaller, Niels Alexander Foit

**Affiliations:** 1 Epilepsy Center, Medical Center – University of Freiburg, Faculty of Medicine, University of Freiburg, Breisacher Str. 64, 79106 Freiburg im Breisgau, Germany; 2 Freiburg Brain Imaging, Medical Center – University of Freiburg, Faculty of Medicine, University of Freiburg, Freiburg im Breisgau, Germany; 3 Department of Neuroradiology, Medical Center – University of Freiburg, Faculty of Medicine, University of Freiburg, Freiburg im Breisgau, Germany; 4 Department of Neurosurgery, Medical Center – University of Freiburg, Faculty of Medicine, University of Freiburg, Freiburg im Breisgau, Germany

**Keywords:** Segmentation, Accuracy, Epilepsy surgery, Temporal lobe

## Abstract

**Purpose:**

Precise segmentation of brain lesions is essential for neurological research. Specifically, resection volume estimates can aid in the assessment of residual postoperative tissue, e.g. following surgery for glioma. Furthermore, behavioral lesion-symptom mapping in epilepsy relies on accurate delineation of surgical lesions. We sought to determine whether semi- and fully automatic segmentation methods can be applied to resected brain areas and which approach provides the most accurate and cost-efficient results.

**Methods:**

We compared a semi-automatic (ITK-SNAP) with a fully automatic (lesion_GNB) method for segmentation of resected brain areas in terms of accuracy with manual segmentation serving as reference. Additionally, we evaluated processing times of all three methods. We used T1w, MRI-data of epilepsy patients (*n* = 27; 11 m; mean age 39 years, range 16–69) who underwent temporal lobe resections (17 left).

**Results:**

The semi-automatic approach yielded superior accuracy (*p* < 0.001) with a median Dice similarity coefficient (mDSC) of 0.78 and a median average Hausdorff distance (maHD) of 0.44 compared with the fully automatic approach (mDSC 0.58, maHD 1.32). There was no significant difference between the median percent volume difference of the two approaches (*p* > 0.05). Manual segmentation required more human input (30.41 min/subject) and therefore inferring significantly higher costs than semi- (3.27 min/subject) or fully automatic approaches (labor and cost approaching zero).

**Conclusion:**

Semi-automatic segmentation offers the most accurate results in resected brain areas with a moderate amount of human input, thus representing a viable alternative compared with manual segmentation, especially for studies with large patient cohorts.

## Introduction

Studying associations between structural brain lesions and observable functional deficits constitutes a well-established approach in neuroscience research [1–3]. Quantitative analysis techniques such as voxel-based lesion-symptom mapping (VLSM) [1, 4] or overlap calculations between lesions and critical structures [5–7] are employed to link structural alterations to behavioral markers [8], hence detecting brain areas critical for a specific behavior. Precise determination of lesioned tissue components in neuroimaging data therefore constitutes a crucial step for these methods [5, 9, 10].

To date, slice-by-slice manual lesion tracing by expert raters remains the gold standard [5, 11, 12]. This approach is considered most precise [3], but also tedious and time-consuming [13] and requires significant experience [14]. Therefore, lesion segmentation constitutes a significant cost factor in neuroimaging research. Semi-automatic methods seek to overcome these limitations [15, 16]. Generally, their approach is based on the assumption that alterations in tissue homogeneity [17], composition, shape [18], or laterality [19] represent lesioned tissue and their identification would only require fine-tuning of parameters by a supervisor [20]. Despite being less laborious than manual tracing, significant amounts of user-interaction are nevertheless required [20].

Recently, several fully automatic methods for lesion segmentation using machine learning (ML) [17, 21, 22] approaches have been proposed. Briefly, ML allows for the design of algorithms that can learn from training datasets and make predictions on new data. ML approaches are usually classified into two broad categories, namely supervised and unsupervised learning [23]. In supervised learning, manually labeled training data is fed to the algorithm to enable categorization of new data—supervision is provided during training [21]. Conversely, unsupervised approaches rely on the recognition of latent patterns within the data to identify groups or clusters [13, 24–28].

In temporal lobe epilepsy (TLE), precise lesion delineation can inform surgical planning [29] and facilitate research on cognitive outcomes [30, 31]. While VLSM constitutes a core component of stroke research [1], its application in TLE has been limited to date. However, with the introduction of selective procedures such as laser amygdalohippocampectomy [32], VLSM in TLE now receives growing interest [7], specifically for the study of memory impairment [33, 34]. Additionally, assessment of lesion volume data can significantly improve image registration and normalization to stereotaxic space [35, 36]. Furthermore, postoperative resection volumes are particularly useful for the assessment of completeness of resection [29] and seizure outcome prediction [37]. Clearly, there is a need to obtain accurate resection volumes from imaging data in TLE. However, to date, no segmentation algorithm specifically designed to investigate neurosurgical resections has been proposed.

In the present study, we therefore sought to determine;

a) whether supervised semi- and fully automatic algorithms each provide satisfying accuracy in resected brain areas compared with manual segmentation;

b) which of the three methods is most time- and cost-efficient and offers the best ratio between quality and cost.

## Methods

### Subjects and imaging data

This retrospective study was approved by the local institutional review board and individual informed consent was waived. High-resolution, T1-weighted magnetization-prepared rapid gradient echo datasets from 27 TLE patients after unilateral anterior temporal lobectomy (ATL) or selective amygdalohippocampectomy (sAHE) acquired with a standard 32-channel headcoil (MPRAGE; TR = 2200 ms; TE = 2.15 ms; flip angle = 12°; 160–176 sagittal slices, voxel size 1 × 1 × 1 mm^3^) on a 3-T TIM Trio clinical MRI scanner (Siemens Healthineers AG, Erlangen, Germany) were retrieved from PACS (Picture Archiving and Communication System). Postoperative imaging was performed during routine follow-up median 3 months after epilepsy surgery (range 1–22 months). Clinical and demographic data of the study group are summarized in Table 1. All statistical analyses were performed with SPSS version 23.0 (IBM, Chicago, IL).

**Table 1:** Demographic data

Patient	Gender	Age at surgery	AoO (years)	Duration (years)	Side of surgery	Type of surgery	Pathology	ILAE seizure outcome—1 year
1	F	34	0.75	33.25	L	ATL	Dual (FCD)	3a
2	M	20	12	8	L	ATL	Dual (FCD)	2b
3	M	53	9	44	L	ATL	Dual (FCD)	3a
4	F	69	17	52	L	ATL	FCD	3b
5	F	32	7	25	L	ATL	FCD	1a
6	F	56	13	43	L	ATL	Dual (FCD)	1a
7	M	38	17	21	R	ATL	HC sclerosis	2b
8	M	50	14.0	36	L	ATL	Dual (FCD)	1b
9	F	16	0.5	15.5	L	ATL	Dual (FCD)	1a
10	M	53	39	14	R	ATL	HC sclerosis	2b
11	M	20	10	10	R	ATL	HC sclerosis	1a
12	F	28	7	21	L	ATL	HC sclerosis	1a
13	M	25	21	4	R	ATL	Dual (FCD)	1a
14	F	55	32	23	R	sAHE	HC sclerosis	1a
15	F	47	3	44	L	sAHE	HC sclerosis	3a
16	M	47	26	21	L	sAHE	HC sclerosis	1a
17	F	46	30	16	R	sAHE	HC sclerosis	2b
18	F	21	20	1	L	sAHE	Dual (ganglioglioma)	1a
19	F	42	32	10	L	sAHE	HC gliosis	1a
20	F	53	10	43	L	sAHE	HC sclerosis	1a
21	M	22	15	7	R	sAHE	HC sclerosis	1a
22	F	35	21	14	L	sAHE	HC sclerosis	1a
23	M	22	19	3	R	sAHE	HC sclerosis	1a
24	M	48	2.0	46	L	sAHE	HC sclerosis	1a
25	F	39	3	36	R	sAHE	HC sclerosis	1a
26	F	50	38	12	L	sAHE	HC sclerosis	4
27	F	35	33	2	R	sAHE	HC sclerosis	1a
Mean	16 F	39.1	16.7	22.4	17 L			63% = 1a; 37% > 1a
Std. dev ±		13.8	11.4	15.4				

*AoO*, age of onset; *F*, female; *M*, male; *L*, left; *R*, right; *Dual*, dual pathology; *FCD*, focal cortical dysplasia

### Preprocessing

All imaging data preprocessing was performed with statistical parametric mapping software (SPM version 12, fil.ion.ucl.ac.uk) running in MATLAB R2016b (The Mathworks, Nattick, USA). After visual inspection of image quality, all T1w images with right hemispheric lesions were flipped to align all pathology to the left.

### Computational platform

Manual, semi-automatic, and fully automatic segmentation was performed on a 3.2-GHz Intel Core i7-6600U platform with 12-GB RAM under Windows 10. MATLAB R2016b and SPM12 were used to run the GNB classifier.

### Manual segmentation

Hand-drawn resection area maps served as ground truth [20]. Delineation was performed by an experienced rater (CS, neuroscience researcher with more than 5 years of experience in manual lesion delineation) blinded to the results of other segmentation methods. The boundaries of the resection areas were hand-drawn on consecutive axial slices with MRIcron (www.mricro.com) in patient space and then automatically filled, resulting in a three-dimensional region of interest (ROI).

### User-guided, semi-automatic segmentation

**Fig. 1:**
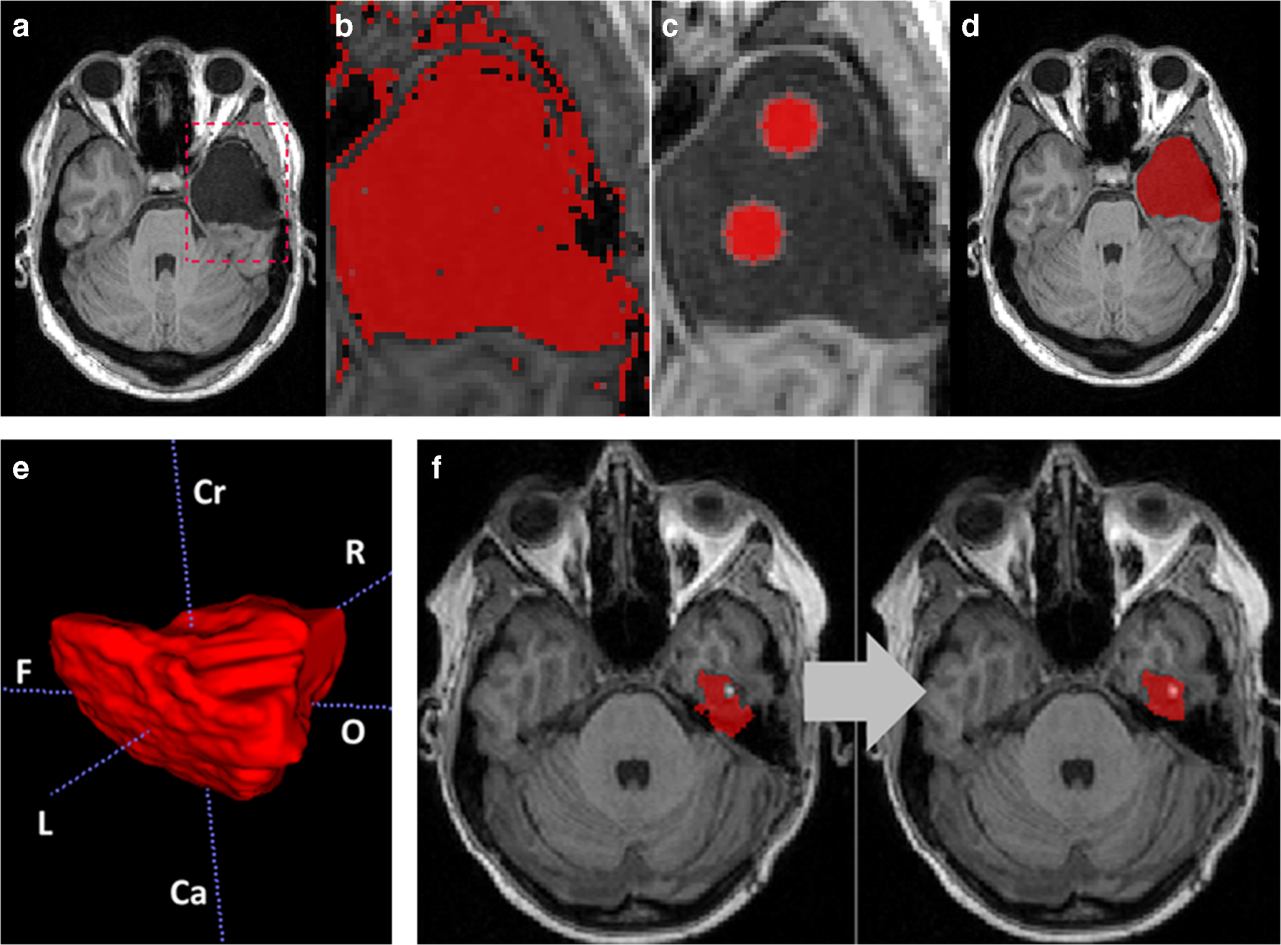
ITK-SNAP segmentation workflow. **a** Region of interest definition. **b** Thresholding. **c** Placement of seedpoints. **d** Quality control. **e** Three-dimensional polygonal lesion model (F = frontal; O = occipital; L = left; R = right; Cr = cranial; Ca = caudal). **f** Manual editing

Semi-automatic segmentation was performed with ITK-SNAP toolbox version 3.6 (further referred to as SNAP) [16]. The “region competition” segmentation approach as well as the active contour evolution algorithm and their implementation in the ITK-SNAP has been previously described in detail [16, 38]. Briefly, the rater defines a segmentation domain to restrict the algorithm to a volumetric ROI. For pre-segmentation, we used the soft-thresholding intensity-based mode [39]: By manually applying a two-sided threshold depending on the intensity range of the ROI (foreground), an intensity grading vector “speed image” is generated to broadly define lesion boundaries. In this speed image, intensity values between the lower and upper thresholds are assigned positive speed values and correspond to parts of the image that have higher probability of representing the ROI rather than the background. Values outside the thresholds map to negative speed values. To initiate geometric active contour segmentation, the rater places at least one seedpoint randomly inside the ROI, which will grow in a way that balances adherence to the speed image with a geometric regularization term [40]. The evolving contour is visualized in real time in 2D slices and evolves either with a fixed step size or continuously until manual termination by the user [41]. We chose to stop the algorithm if there was no further visible propagation in the image over 5 seconds or if the rater decided that the evolving segmentation began to leak outside the boundaries of the ROI. This is a common approach other studies described before [42]. Finally, ROIs were inspected for quality and manually edited in two cases (shown in Fig. 1f): Firstly, if active contour segmentation bled into CSF space, manual editing was performed using the paintbrush tool. Secondly, if parts of the resected brain area, e.g., blood debris, were not identified as part of the ROI, we used the paintbrush with the interpolation module that allows to trace a structure in just a handful of slices, with the algorithm filling in the intermediate slices [43]. This segmentation method was used as it requires only minimal user-interaction and reliable results can be obtained from single-modality MRI. In this study, an expert rater (NAF, neuroscience researcher with more than 10 years of experience in lesion segmentation) performed the segmentation with ITK-SNAP blinded to the results of manual segmentation. Figure 1 illustrates individual steps of the semi-automatic segmentation process.

### Fully automatic segmentation

The lesion_GNB software package by Griffis and coworkers [44] employs a supervised approach based on Gaussian Naïve Bayes (GNB) classification for the delineation of chronic stroke defect zones. To provide ground truth for classifier training, manual segmentation of T1w MRI data of 30 patients with chronic stroke defect zones was performed by the authors [44]. Briefly lesion_GNB relies on probabilistic tissue segmentation and image algebra to create feature maps encoding information about missing or abnormal tissue classes. The GNB classifier was trained on the ground-truth manually delineated lesions and validated using a leave-one-out cross-validation approach. It does not require a control group. By using default SPM processing routines, transformation parameters for normalization to Montreal Neurological Institute (MNI) space are created during analyses [44].

### Spatial similarity analyses

Most automated segmentation routines operate in algorithm-specific stereotaxic space, which may not correspond to MNI space [45]. However, studies investigating larger patient cohorts will ultimately require normalization to make group inferences. To avoid potential bias by comparing inverse-normalized (lesion_GNB) to native-space (manual, ITK-snap) results, we chose to perform spatial similarity analysis in common space. All ROIs were therefore normalized to standard MNI space with normalization parameters obtained during the lesion_GNB preprocessing of individual subjects.

Degrees of overlap between the different lesion segmentation techniques referenced against manual tracing results were evaluated voxel-by-voxel with the Dice similarity coefficient (DSC), a robust metric of both overlap and reproducibility [46]. The DSC is calculated according to $\mathrm{DSC}=\frac{2\left(\mathrm{X}\cap \mathrm{Y}\right)}{\left(X+Y\right)}$. In case of partially overlapping image volumes, i.e., reference (*X*; manual tracing) and predicted volume (*Y*; semi-/fully automatic approach), the DSC ranges from 0 (no overlap) to 1 (total congruence), with larger values indicating better performance [42]. DSCs ranging above 0.6 are good, values above 0.7 are considered high, and values exceeding 0.8 are excellent [13, 20, 44].

Although robust and straightforward to interpret, the DSC does not weigh false-positive or negative results in any way. This becomes particularly relevant in complex structures such as surgical lesions, where boundary agreement between segmentations might be of interest [47]. Therefore, we chose to supplement the analysis with a measure of spatial distance, i.e., the average Hausdorff distance (aHD). The aHD represents the maximum distance of all voxels in one dataset to corresponding voxels in the test set [48], thus quantifying similarity or discrepancy between two given structures [49]. The aHD between two point sets X and Y is defined as aHD(*X*, *Y*) = max(*d*(*X*, *Y*), *d*(*X*, *Y*)) where *d*(*X*,*Y*) is the directed aHD that is given by $d\left(X,Y\right)=\frac{1}{N}\sum \limits_{x\in X}\underset{y\in Y}{\min}\left\Vert x-y\right\Vert$ with reference (*X*; manual tracing) and predicted volume (*Y*; semi-/fully automatic approach). All distances are calculated in voxel with smaller values indicating better performance.

As the results were not normally distributed, they were compared using non-parametrical Wilcoxon signed-rank test, respectively. In order to assess the relation between performance of the approaches and the resection area size, a median split by lesion volume (based on manual segmentation) was performed. With a Mann-Whitney *U* test, the difference of the metrics of the two groups (small/large lesions) within each approach was assessed and a Wilcoxon signed-rank test was used to compare the approaches within each group. Correlations were assessed with the non-parametrical Spearman’s rank correlation.

### Volumetric analyses

Percent lesion volume differences (in ml) between manual and (semi-)automated segmentation results were also evaluated. Percent volume difference (PVD) was calculated according to $\mathrm{PVD}=\left[\frac{\mathrm{Vreference}-\mathrm{V}\left(\mathrm{semi}-\right)\mathrm{automatic}}{\mathrm{Vreference}}\right]\times 100$; with *V*_reference_ and *V*_(semi-)automatic_ representing volumes of manual and (semi-)automatic resection maps, respectively. Since resection volumes were not normally distributed, a Wilcoxon signed-rank test was used for comparisons. We also assessed differences between large and small resections in the PVD within each approach with the Mann-Whitney *U* test.

### Effort and cost

The labor time (corresponding to human input) in minutes required from the beginning of the segmentation to saving the final ROI was recorded for each method. The time required for loading the image into each program was similar, so we did not take this step into account. As in the fully automatic approach no human input was necessary after loading the images, labor time therefore resulted in 0.00 min/subject. For manual and semi-automatic segmentation, the labor time was normally distributed, as assessed by the Shapiro-Wilk test (*p* > 0.05). The difference in time expenditure between the manual and semi-automatic approaches was compared with a paired-samples *t* test. To compare the labor time of the manual and semi-automatic to the fully automatic approach (0.00 min/subject), a one-sample *t* test was used, respectively.

Because we only used open-source software and the available computational infrastructure, the total method cost in this study was mainly composed of personnel cost based on the salary rates of the German Research Foundation for 2019 (available at www.dfg.de/formulare).

## Results

### Patient characteristic

Data were acquired from 27 patients (16 females, 11 males), who underwent unilateral ATL (13 patients) or sAHE (14 patients) for pharmacoresistant TLE (17 left-sided, 10 right-sided). The mean age at surgery was 39.1 years (range 16–69), with a mean age at epilepsy onset of 16.7 years (range 0.5–39) and a mean disease duration of 22.4 years (range 1–52). Approximately 63% of the patients were seizure-free (Engel IA) after surgery. The predominant pathology was hippocampal sclerosis (16 patients) followed by dual pathology (i.e., hippocampal sclerosis combined with focal cortical dysplasia; 8 patients). Detailed demographical and clinical data are given in Table 1.

### Accuracy

**Fig. 2:**
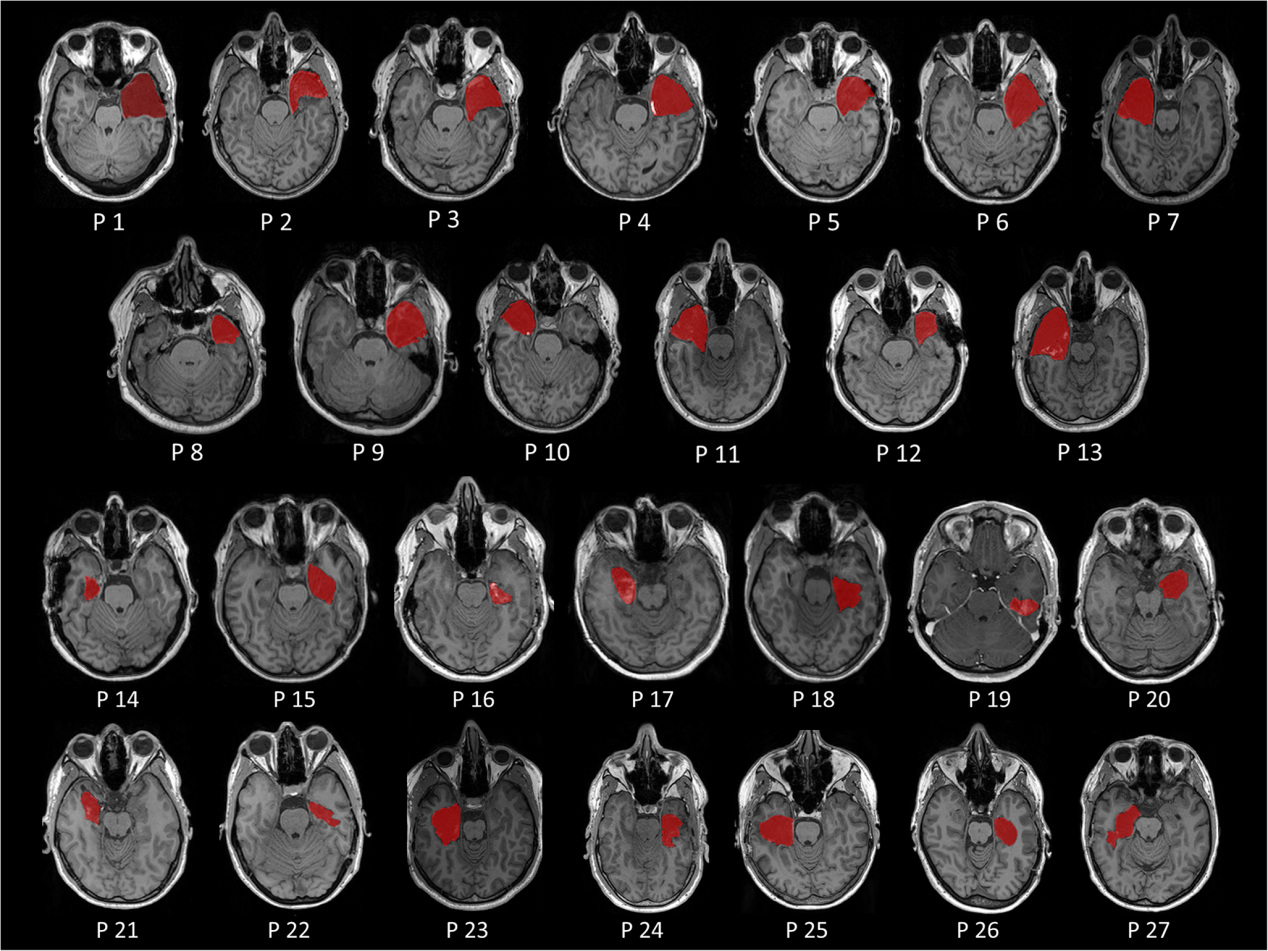
Representative axial slices of the resected brain areas with superimposed manual segmentations (ATL: patients 1–13; sAHE: patients 14–27)

Manual segmentation is the gold standard and serves as ground truth for the other methods. Figure 2 illustrates 27 manually segmented resection areas, overlaid on the respective T1w image.

**Fig. 3:**
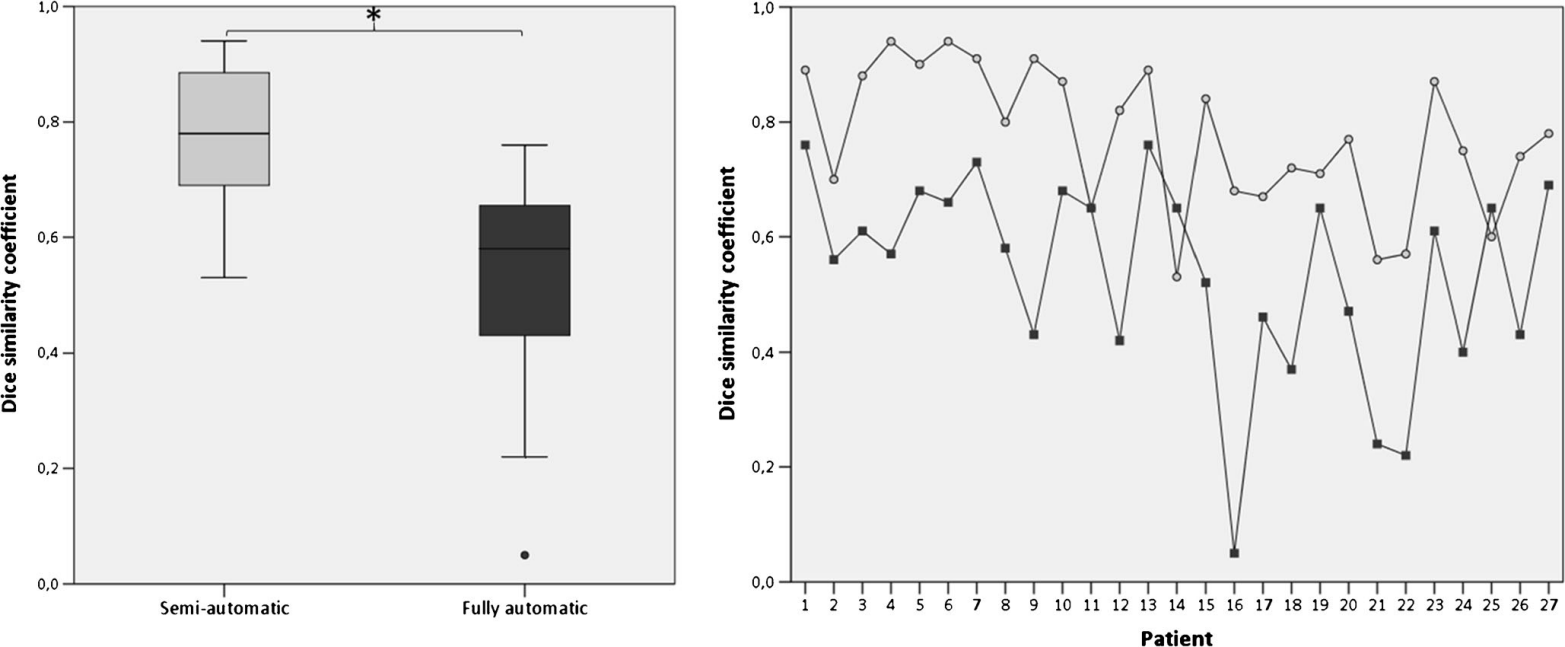
DSC for semi-automatic (light) and fully automatic (dark) approach (*signif. *p* < 0.001)

The median DSC for the semi-automatic approach relative to the manual segmentation was 0.78 (range 0.53–0.94), indicating a high overlap. For the fully automatic method, the median DSC was 0.58 (range 0.05–0.76). At *p* < 0.001 (*z* = − 4.332), the results of the semi-automatic approach were significantly better than those of the fully automatic (Fig. 3).

**Fig. 4:**
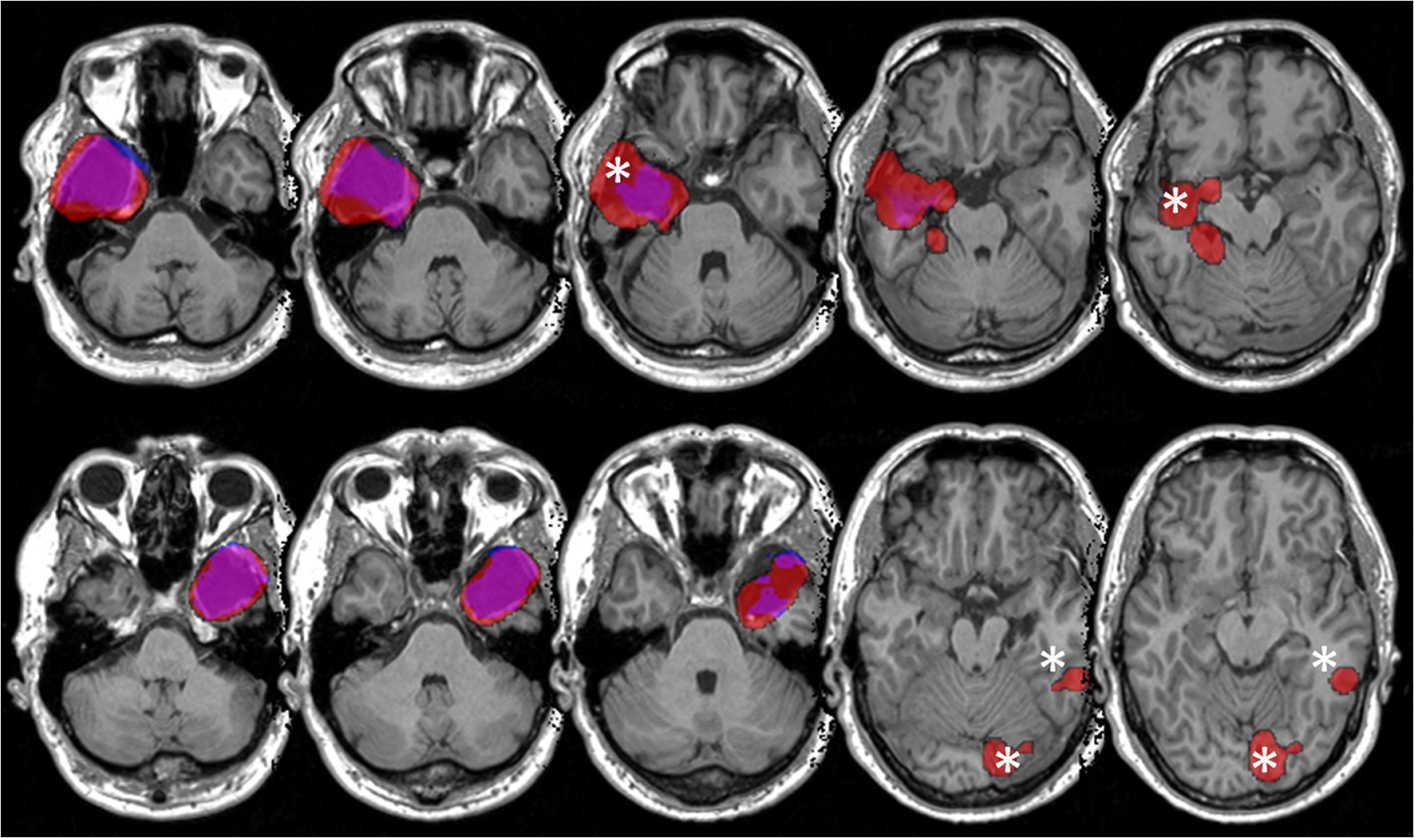
Individual false-positive results in automatic processing. GNB classifier results (red) are superimposed onto manual segmentation (blue). Areas of congruence are purple. Asterisk (*) indicates areas of false-positive values

**Fig. 5:**
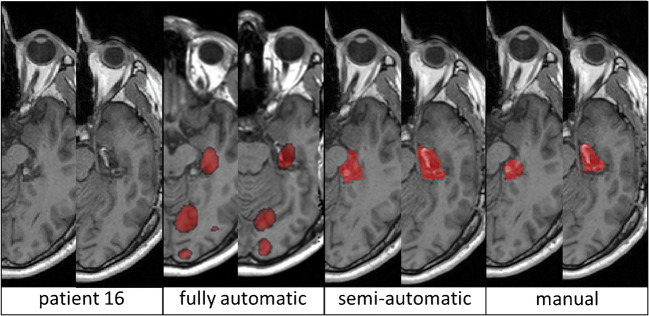
Individual performance of all approaches in patient 16 (axial slices)

**Fig. 6:**
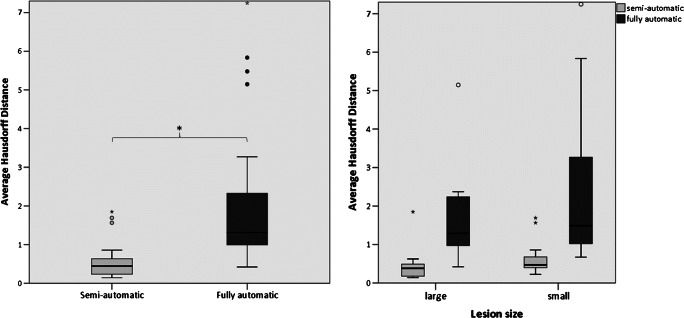
Averaged Hausdorff distance for semi-automatic (light) and fully automatic (dark) approaches (*signif. *p* < 0.001)

As shown in the right panel in Fig. 3, the fully automatic segmentation method performed worse in all cases except three, with several false positive results as shown in Fig. 4. The GNB classifier failed to identify one resection area (P16, DSC 0.05) and performed poorly in another two cases (P21, P22). Figure 5 shows the individual performance of all approaches in patient P16. Even if this case was excluded, there would still be a highly significant difference (*p* < 0.001, *z* = − 4.239) between the fully automatic (median DSC 0.60) and the semi-automatic (median DSC 0.79) approach. In terms of spatial distance, the semi-automatic approach (median aHD 0.44, range 0.14–1.85) similarly outperformed (*p* < 0.001) the fully automatic approach (1.32, range 0.42–7.25; Fig. 6—left panel).

To assess the impact of actual lesion size on accuracy, all cases were dichotomized into groups by their median resection volume obtained from manual delineation (median = 17.920 ml), resulting in 14 large (mean volume 26.02 ± 8.06 ml, range 17.92–48.23 ml) and 13 small resection areas (mean volume 7.39 ± 3.08 ml, range 2.22–12.36 ml).

**Fig. 7:**
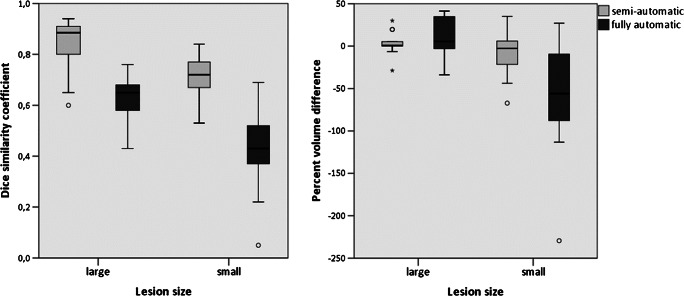
DSC (left) and PVD (right) of each approach in large and small lesions

As shown in Fig. 7 (left panel) and Table 2, larger resections were associated with significantly better DSCs than smaller resections for both methods (*p* < 0.05). Additionally, resection size was significantly correlated with the DSCs in both semi- and fully automatic approaches (*r*_s_ = 0.71, *p* = 0.001 vs. *r*_s_ = 0.54, *p* = 0.004). Comparing spatial similarity with aHD, we found no significant difference between the performance in small and large resection areas in both approaches, respectively (Fig. 6, right panel and Table 3).

**Table 2:** Median DSCs of semi- and fully automatic segmentations in small and large lesions

Method	Lesion size	Median DSC	Sign.
Semi-automatic	Large	0.89 (SD ± 0.11, range 0.60–0.94)	*p* < 0.05, *z* = − 2.962
	Small	0.72 (SD ± 0.10, range 0.53–0.84)	
Fully automatic	Large	0.65 (SD ± 0.09, range 0.43–0.76)	*p* < 0.05, *z* = − 3.039
	Small	0.43 (SD ± 0.18, range 0.05–0.69)

**Table 3:** Median aHDs of semi- and fully automatic segmentations in small and large lesions

Method	Lesion size	Median aHD	Sign.
Semi-automatic	Large	0.38 (SD ± 0.45, range 0.14–1.85)	*p* > 0.05
	Small	0.47 (SD ± 0.47, range 0.22–1.69)	
Fully automatic	Large	1.29 (SD ± 1.22, range 0.42–5.15)	*p* > 0.05
	Small	1.49 (SD ± 2.21, range 0.67–7.25)	

The accuracy of the semi-automatic approach however significantly outperformed the fully automatic approach irrespective of the resection size (*p* < 0.001 for DSC, *p* < 0.05 for aHD).

The median PVD with reference to manual segmentation was 0.04 (SD ± 21.57; range − 67.18–35.11) for the semi-automatic and − 3.13 (SD ± 61.75; range − 229.32–41.37) for the fully automatic approaches. There was no significant difference between the approaches (*p* > 0.05). In small resections, the PVD of the semi-automatic approach was significantly better than the PVD of the fully automatic approach (*p* < 0.05, *z* = − 2.691), whereas in large resections, there is no significant difference (Table 4). The PVDs of the semi-automatic approach did not reveal a significant difference regarding small or large resections, while in the fully automatic approach, the median PVDs significantly differed between the groups (*p* < 0.05, *z* = − 3.057), with better performance in large resections.

**Table 4:** Median PVDs of semi- and fully automatic segmentations in small and large lesions

Method	Lesion size	Median PVD	Sign.
Semi-automatic	Large	0.72 (SD ± 13.71, range − 28.91–29.96)	*p* > 0.05
	Small	− 2.75 (SD ± 27.26, range − 67.18–35.11)	
Fully automatic	Large	5.44 (SD ± 23.63, range − 33.97–41.37)	*p* < 0.05, *z* = − 3.057
	Small	− 55.88 (SD ± 70.33, range − 229.32–27.04)	

### Effort and cost

The average time needed for manual segmentation was 30.41 min/subject (SD ± 6.43, range 17–50 min). User-guided semi-automatic lesion segmentation took an average of 3.27 min/subject (SD ± 0.96, range 1.33–5.53 min), including manual correction for inaccuracies. The differences in effort between manual and semi-automatic as well as manual and fully automatic or semi-automatic and fully automatic approaches were highly significant at *p* < 0.001, respectively.

Importantly, it must be mentioned that the fully automatic approach required a certain amount of processing time on our computational environment, which was 13.41 min/subject (SD ± 2.14, range 9–19). This was however irrelevant for cost calculation, because this step did not require supervision and the main financial burden stems from personnel cost.

Then, we made a digression into the cost factor of this study. For manual segmentation, an expert rater was needed, whereby at least a postdoctoral researcher is meant (standard salary 6000€/month, 34.62€/h). Hence, the mean cost was 17.64€/per subject. To ensure an optimal outcome of the manual corrections, an expert rater would be equally required for the semi-automatic approach. For this approach, an average of 1.90€/subject had to be invested. The fully automatic segmentation with GNB required no human input for the image segmentation. In that case, mean cost was approaching zero, if image loading steps are disregarded.

## Discussion

This study compared manual to semi- and fully automatic methods for segmentation of resected brain areas on high-resolution T1w images. One of the most important questions concerning medical image segmentation is accuracy. We investigated quality and validity of the applied methods using manual segmentations as ground truth. Measures used were the DSC as a global measure of overlap, the aHD as a metric of spatial distance, and PVD for volume agreement, because our intention was to focus on few but relevant parameters which are straightforward to obtain and interpret. The DSC constitutes a robust measure of overlap and has been widely used in the validation of other methods for lesion segmentation [17, 42, 44]. It takes both false positives and false negatives into account and therefore represents a robust performance indicator.

Analyses were completed by the aHD, which is especially recommended when the accuracy of the boundary delineation of the segmentation method is of importance, as it is the case in TLE surgery [47]. However, conventional HD is sensitive to and over-penalizes outliers, which are very likely in the comparison with surgical segmentations [47]. Average HD overcomes these limitations and is therefore particularly well-suited for anatomical image analyses [50]. Irrespective of the used metric, results must be still interpreted with caution—despite generally favorable global aHD, there could still be considerable local disagreement within a complex structure [49].

The semi-automatic approach implemented in the ITK-SNAP outperformed the fully automatic method independent of lesion size and achieved excellent accuracy (median DSC of 0.78, median aHD 0.44). The high signal intensity contrast between the surgical defect zone and brain tissue as well as the well-defined resection boundaries may have contributed positively to this good performance.

Corroborating previous studies, we found a strong influence of individual lesion size on the DSC [20]. Expectably, the segmentation performance using either approaches was significantly better in larger resections, as the DSC is sensitive to the size of the segmented area [49]. Segmentation errors were indeed mostly located at the boundary and could therefore bias the analysis towards larger segments. Results of the spatial distance analysis support this hypothesis, because it is independent of the size of the segmented area [51].

Focusing on the cases with limited performance of the semi-automatic approach (7 patients with a DSC < 0.7), we found interesting commonalities beside lesion size. In three patients (P14, P16, P25) it was difficult to separate the resected area from the enlarged inferior horn of the lateral ventricles. Three resection cavities (P16, P17, P21) were inhomogeneous, and the resection boundaries were not clearly apparent. Segmentation with the ITK-SNAP is relatively robust and in these cases even turned out to be significantly more reproducible than manual segmentation [16]. It can be performed by any rater with experience in manual segmentation [41]. Additionally, placement of the seeds in the ROI does not represent a crucial step per se, as the evolving contour expands in all directions in regions where the speed function is positive and contracts where the speed function is negative. We decided to use the ITK-SNAP due to its simple handling, the evolving visualization of the 3D volume in real time, and the option to post-process the segmentation using integrated 3D manipulation tools.

The automatic algorithm delivered less satisfactory results (median DSC 0.58, median aHD 1.32) and did not detect the resection in a single subject, where it detected only parts of the resected area (Fig. 4). It should however be taken into consideration that the resected area was difficult to segregate from surrounding tissue, as it contained blood debris [52]. This is in line with previous studies which demonstrated reduced precision when tissue signal intensities were similar or the target structure itself was altered [15, 21].

This signifies a clear advantage of the semi-automatic approach, as the manual interaction described above ensures satisfying results even in cases were other methods might fail. It has however to be emphasized that the GNB classifier was trained on stroke lesions, and while both surgical resection cavities and resorbed lesioned tissue in chronic stroke will ultimately contain CSF, there are still differences in signal intensity. Nevertheless, no segmentation algorithm specifically designed for neurosurgical resections has been proposed to date, necessitating use of established methods.

Notwithstanding expectable limitations, selection of the lesion_GNB was driven by several potential advantages: It operates on unimodal T1w data, requires no control population or arbitrary thresholding, and integrates with SPM, utilizing default segmentation and normalization routines [44]. This precluded consideration of other algorithms from the ischemic stroke lesion segmentation (ISLES) challenge [53], as they require either multispectral data [41, 42] or healthy control groups [13], which might not be readily available in all research environments. While our results revealed reduced accuracy, implementation of TLE-specific training datasets into the lesion_GNB would clearly improve lesion detection. This was however beyond the scope of this study and will be addressed in future work.

As a second point, we assessed practical aspects of the different segmentation methods. Regarding practical implementation in research environments, we relied on freely available software and standard computational hardware. As previous studies emphasized, manual segmentation is extremely tedious and, depending on lesion complexity, very time-consuming and thus expensive [13, 20, 54]. Manual segmentation in our study required an average human input of 30.41 min/subject, mainly due to the uniformity of TLE resections. Manual tracing furthermore requires sound anatomical knowledge, at least at the level of postdoctoral research. For larger cohorts, manual segmentation would therefore implicate a tremendous amount of manual work and cost.

The semi-automatic method requires similar expertise; it was however significantly faster compared to manual tracing (3.27 min/subject), resulting in a cost reduction by 15.74Euro per subject. In this regard, fully automatic segmentation methods could offer even better cost-effectiveness. They are less time-consuming and do not require an expert as they can be applied by individuals with limited experience [44]. Interaction during processing is minimal, resulting in cost per subject approaching zero. The lesion_GNB algorithm performed well on standard computers (13.41 min/subject), whereas availability of high-performance computational equipment would further increase throughput.

## Conclusion

Our findings suggest that semi-automatic methods are currently most efficient for the segmentation of surgical resections. They offer the best compromise between precision and effort, which is particularly relevant for the evaluation of larger cohorts. Its superior accuracy compared to the automatic method proved that human input can further improve computerized segmentations.

## Limitations

No formal assessment of inter-rater reliability in the manual or semi-automatic approaches was attempted here as this was extensively explored in previous studies [55]. Although the cohort was small, the sample size was sufficient to demonstrate significant differences between the approaches. Moreover, although intra-class correlation coefficients (ICC) are often used to compare segmentation techniques, the ICC strongly depends on sample size and distribution of subjects, especially in smaller cohorts [56], thus precluding use of ICC as a measure of accuracy in our study.
